# An automated titration system

**DOI:** 10.1155/S1463924681000564

**Published:** 1981

**Authors:** T. Pap, M. Molnár, J. Inczédy

**Affiliations:** Institute of Analytical Chemistry University of Veszprem Hungary


Nevertheless there are two fundamentally different ways of identifying sets of data. First, the cumulative collection of test results may be checked for plausibility. Mislabelled results will be recognised if they differ greatly from those recorded earlier. The method, however, is not very effective as Sheiner *et al* [16] have shown. A Delta check method, controlled by a computer, discovered only 50% of mislabelled specimens. On the other hand only 10% of the data suspected of being mislabelled were in fact wrongly identified.

The second approach is based on the biochemical individuality characterising a healthy, or a sick person unequivocally, if a sufficient number of tests is performed. Young and his co-workers [17] have recently demonstrated that the identity is reflected by multivariate data of biochemical test profiles. Profiles of individual samples were graphically displayed as computer-drawn faces and they conclude from their experience the following: "Discriminant functions can be used to detect mislabelled specimens in research studies and potentially in the clinical laboratory to detect misidentified samples from patients". Obviously this will not be a practicable method for sample identification in our laboratories. But reflecting on the coherence of individuality and identification may be worth while.

REFERENCES

[1] Haeckel, R. (1980), Rationalisierung des medizinischen Laboratoriums GIT Verlag Darmstadt 2nd. Ed.

[2] Rubin, M. (1969), VII Int Congr Clin Chem, Geneva Diskussionsbeitrag.

[3] Keller, H. and Richterich, R. (1972), VIII World Congress of Anatomic and Clinical Pathology Munich 12–16 Sept, Excerpta Medica Amsterdam Internat Congr Series No 285, 183-189.

[4] Richterich, R. and Greiner, R. (1971), *Z Klin Chem Klin Biochem,* 9, 187-193.

[5] Hille, W. and Knaus, M. (1973), *GIT Fachz Lab,* **17**, 1255.

[6] Knedel, M. (1971), Siemens Druckschrift Nr. MC 50/1016.

[7] Sher, P. P. and Gambino, S. R. (1970), *Adv Autom Analysis,* **1**, 63-65, Thurmann Ass Miami, Florida.

[8] Rappoport, A. E., Gennaro, W. D., and Constandse, W. J. (1968), *Modern Hospital,* **110**, 100-103.

[9] Borner, K. and Klein, E. (1969), *Z Klin Chem Klin Biochem,* **7**, 185-188.

[10] Jentzsch, D. (1969), Probenidentifizierung bei klinisch-chemischen Analysen. Berichte zur klinischen Chemie. Perkin Elmer & Co. Ueberlingen/Bodensee Heft 1.

[11] Dudek, J., Roka, L. and Michel, H. (1974), *Aerztl Lab,* **20**, 430-436.

[12] Laue, D. (1977), The Bar-code: A universal system of identification in the medical laboratory. Birmingham 2nd Internat. Conference, Sept 12–16.

[13] American Blood Commission (1976), CCBBA special issue: August 1976, New blood pack label being tested. Am Blood Commission, Arlington, Va 22209.

[14] Michel, H. (1977), *Biotechn Umschau,* **1**, 23.

[15] Reviewed by Keller, H. (1979), *J Clin Chem Clin Biochem,* **17**, 57-64.

[16] Sheiner, L. B., Wheeler, L. A. and Moore J. K. (1979), *Clin Chem,* **25**, 2034-2037.

[17] Robertson, A. E., Steirteghem, A. C., Byrkit, J. E. and Young, D. S. (1980), *Clin Chem,* **26**, 30-36.

### An automated titration system

**T. Pap, M. Molnár and J. Inczédy**

*Institute of Analytical Chemistry, University of Veszprem, Hungary*

### Introduction

The automation of titrations has a long history, but since the introduction of digital computers into laboratories interest in and the possibilities of automation have significantly increased. Johansson [1,2] developed software packages for the calculation of concentration and equilibrium constant data from acid-base titration curves. Leggett [3] connected an automatic burette to a microprocessor and controlled the addition of the titrant to the solution. Similar systems were developed by Firstenberg [4], Betteridge *et al* [5], Nowogrocki [6] and recently Gampp, Maeder, Zuherbükler and Kaden [7].

This system is used for the titration of acid mixtures. The protonation constants of the conjugated bases were determined by additional titration of the solution of the single acids.

In the course of the work reported here, the automatic titration system was constructed from commercially available units. The system could be used to carry out titrations of mixtures and to calculate equivalency points, equilibrium constants and to print out results and record the titration curves.

### Titration system

The block scheme of the titration system can be seen in Figure 1. The control unit is a desk top calculator (EMG 666, Hungary) of 8 KB capacity. It is connected to a mosaic printer (EMG 896, Hungary), a plotter (Videoton NE-2000-666, Hungary), an impulse burette (Metrohm 111, Switzerland) and a digital voltmeter (MIKI, Type 1747, Hungary). A precision pH-meter (Radelkis OP 208, Hungary) and a combined glass-calomel electrode (Radelkis OP-8083, Hungary) were used for the acid-base titrations. The analog signal of the pH-meter was fed to the digital voltmeter and the digital signal of the voltmeter was transferred to the calculator via an interface (MIKI, Type 2702, Hungary). Another interface (EMG 79845, Hungary) was used to connect the impulse burette and the calculator.

The operation of the system for acid-base titrations is as follows. During the titration the calculator calculates the volume of the next titrant fraction from the value of the pH change using a given mathematical function and gives the command to the burette for a proper number of impulses. 0.001 ml titrant is delivered at each impulse. After the addition of the titrant, the solution is allowed to equilibrate and a command is given to store the measured pH value. The titration is continued until the burette is empty (10 ml). Finally, the evaluation process starts.

As mentioned earlier, there is an interface unit between the calculator and the burette which can be seen in Figure 2. The original unit was extended with a home-made coupling board to facilitate the above mentioned operations. This board consists of an address decoding and a command receiving section. When the command comes from the calculator for an impulse the "BUS IFC A" gives the address of the assigned peripheral. When the address is recognised and resignalled in the decoding section of the coupling board, the "BUS IFC A" directs the valid message to the proper bus line. Thus, the command receiving part of the coupling board closes the electric circuit of the impulse burette and in this way, an impulse or a series of impulses is delivered.

### The software

The block scheme of the controlling program can be seen in Figure 3. First, a command is given to measure the pH of the solution. Approximately 30 measurements are averaged in every 5 seconds. After the measurement, a $V_k$ volume portion of the titrant is added. The titrant volume fractions are summed and when the sum is equal to the total volume of the burette, $V_b$, the titration is stopped and the evaluation process as well as the recording of the titration curve are started. As long as the sum of the volume fractions added is less than $V_b$, the calculated new titrant fraction is added. After a certain period T, the pH is measured followed by a second period of time T and another pH measurement. If the difference between the two successive measurements is less than $\epsilon$, the second value is accepted and stored. If the difference is larger than $\epsilon$, another period of time T is allowed to pass and the measurement is repeated until the prescribed conditions prevail and then the last pH value is accepted.

In the next step, the actual slope of the titration curve and the next volume fraction of the titrant derived from the slope are calculated according to the following equation:

$$V_i = V_k \exp\left[-3\left(\frac{\Delta pH}{\Delta V}\right)_i\right] \qquad (1)$$

If $V_i < 0.001$ ml, then 0.001 ml will be delivered and the summation step follows.

During the titration of strong acids, or the titration of a mixture of a strong and weak acids, the calculated actual $\Delta pH/\Delta V$ values can be printed out and the equivalency points can be easily determined. The titration process can be stopped at the maximum value and the concentrations can be calculated when the difference of the logarithms of the

**Table 1. Determination of formic and acetic acid in each other's presence by potentiometric titration**

| Acid mixture | Added mmol | Found mmol | Difference % |
|---|---|---|---|
| Formic acid + | 0.4874 | 0.4819 | -1.13 |
| acetic acid | 0.5133 | 0.5188 | +1.07 |

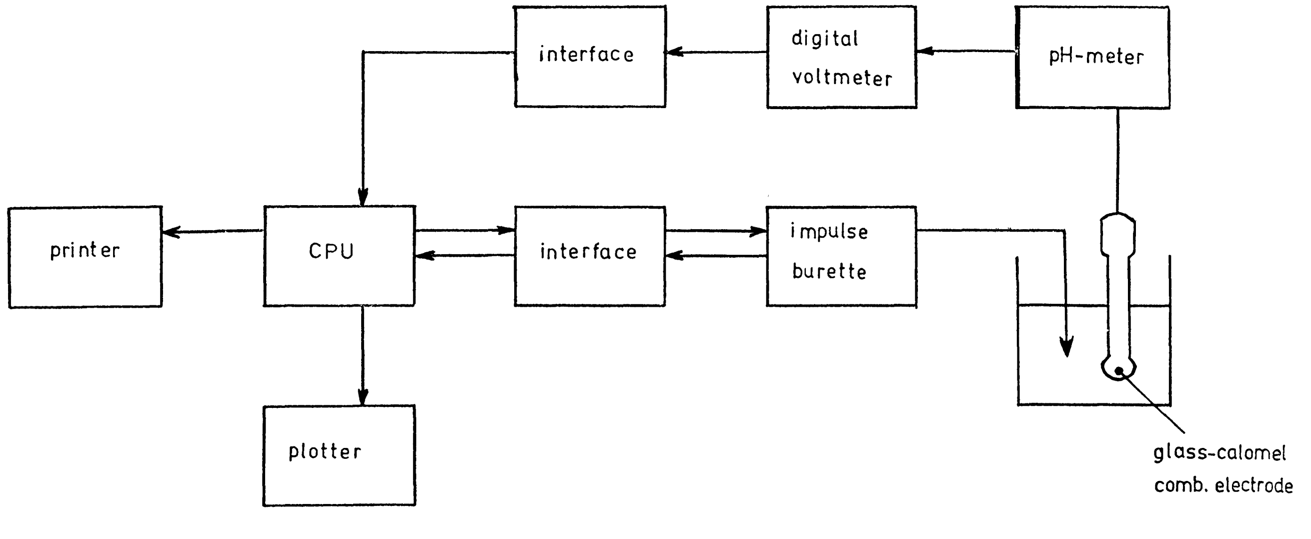

*Figure 1. Schematics of the titration system*

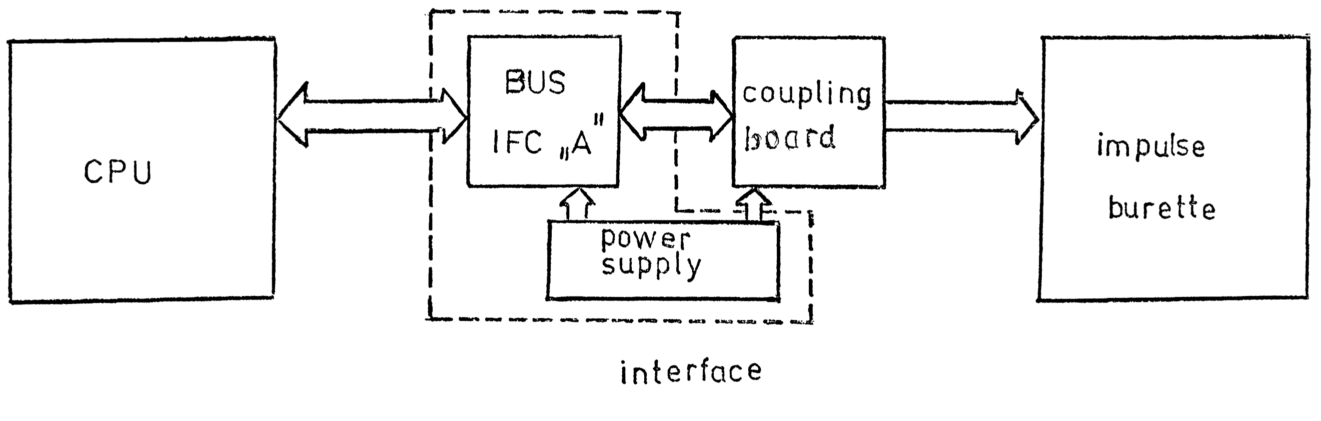

*Figure 2. Schematics of the interface*

constants is greater than 4. Using another program the protonation constants of the acids can also be calculated.

To calculate the amounts of two acids with closely similar protonation constants, the usual methods – as discussed above – cannot be used. This is the case when a mixture of formic and acetic acid is to be titrated (Figure 4).

The following iteration procedure was used to calculate the equivalency points and also the protonation constants of the conjugated bases.

If two weak acids are present the following equation is valid [7]:

$$\frac{V_i C_t}{V_o + V_i} = \frac{C_t \frac{V_a}{V_o + V_i}}{1 + [H^+]_i K_1} + \frac{C_t \frac{V_m - V_a}{V_o + V_i}}{1 + [H^+]_i \; K_2}$$

$$+ \frac{k_v}{[H^+]_i} - [H^+]_i \qquad (2)$$

where

- $V_i$ volume of the titrant after the i-th addition (ml)
- $V_o$ original volume of the titrant (ml)
- $C_t$ concentration of the titrant (mol/dm$^3$)
- $[H^+]_i$ hydrogen ion concentration after the i-th addition (mol/dm$^3$)
- $K_1$;$K_2$ protonation constants of the conjugated bases of the weak acids ($K_1 < K_2$)
- $V_a$ volume of the titrant at the first equivalency point (ml)
- $V_m$ volume of the titrant at the second equivalency point (ml)
- $k_w$ ion product of water
- i number of the titration point

There are four unknowns in equation (2): the two volumes belonging to the equivalency points ($V_a$, $V_m$) and the two constants ($K_1$, $K_2$). When the logarithms of the constants of the acids are smaller than 7, the second equivalency point can be calculated with good precision from the data points of the titration curve using numerical derivation. However, an iteration method has to be used for the calculation of the $V_a$ and $K_1$, $K_2$ values. The values should be varied until the square sum of the differences of the points of the calculated and the experimentally derived curves reach a minimum value.

### Experimental

Stock solutions of hydrochloric, formic and acetic acids were prepared. Their concentrations were determined individually using an 0.1083 M potassium hydroxide standard solution. The initial volume of the titrated solutions were kept constant, $V_o$ = 70 ml. The titrations were carried out at 25 ± 0.5°C.

The titration of the sample solution was started when the pH change in 5 minutes was less than 0.005. During the titration the pH values were calculated from the measured potential differences using the equation obtained by calibration. The calibration was repeated daily. During one day, however, it was not necessary to change the constants of the calibration function.

### Results and discussion

The titration curve obtained by titrating a mixture of formic and acetic acid can be seen in Figure 4. Using the data points of the titration curve, the following calculation procedure was used. Firstly, the second equivalency point and the corresponding titrant volume ($V_m$) were calculated from the position of the inflection point. Using equation (2), the $V_a$ value (volume of the titrant corresponding to the amount of formic acid) was calculated from the points of the titration curve taken from the range of 15-85% titration degree. The

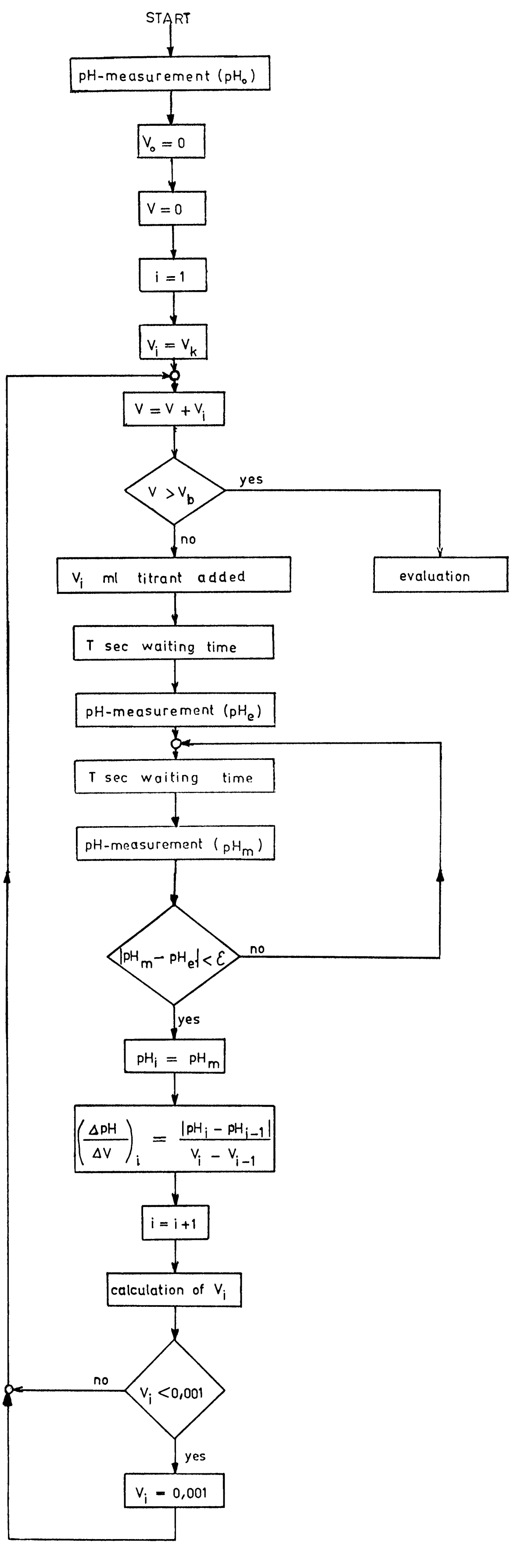

*Figure 3. Schematics of the program system used to control the titration*

calculations were carried out with log $K_1$ and log $K_2$ values taken from the literature [10], then repeated with new values of 0.1 units larger and smaller than the original ones. Thus, the $V_a$ values were calculated from the data points in four different cases and averaged individually. Using the four average values four titration curves were calculated along with the sums of the squares of the deviations from the experimentally obtained ones. In a second step log $K_1$, or log $K_2$ was changed in such a manner that the sum of the squares became lower. If the sum of the squares was found to be greater again, the calculation was returned to the former step. The cycles were repeated with new log $K_1$ and log $K_2$ values until the minimum value for the sum of the squares was achieved. The log $K_1$ and log $K_2$ values found were 3.47 and 4.55 respectively. The calculated quantities of formic and acetic acids are given in Table 1. It can be seen that the accuracy of the determination – considering the fundamental difficulties – is acceptable.

Using the iteration method, there is no need for the extra titration procedures of the single acids and for the determination of the constants. This is especially advantageous when the ionic strength of the sample solution is not known. As a matter of fact, when the titrations of the single acids are carried out at different ionic strengths an uncontrollable error can be introduced into the calculations.

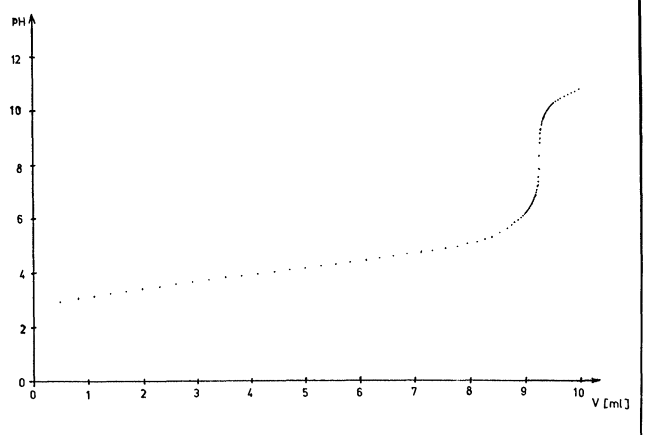

*Figure 4. Titration curve of a solution containing formic and acetic acid. Titrant: carbonate free potassium hydroxide: 0.1083 mol/dm$^3$*
*$V_m$ = 9.29 ml; $V_a$ = 4.45 ml*

REFERENCES
[1] Johansson, A. and Johansson, S. (1978), *Analyst,* **103**, 305-316
[2] Johansson, A. and Johansson, S. (1979), *Analyst,* **104**, 601-612
[3] Leggett, D. J. (1978), *Anal Chem,* **50**, 718
[4] Firstenberg, S. and Malmvig, H. (1978), *American Laboratory,* 2, 119-122
[5] Betteridge, D., Dagless, E. L. and David, P. (1976), *Analyst,* **101**, 409-420
[6] Nowogrocki, G., Cannone, J. and Wozniak, M. (1979), *Anal Chim Acta,* **112**, 185-192
[7] Gampp, H., Maeder, M., Zuberbukler, A. D. and Kaden, T. A. (1980), *Talanta,* **27**, 313-318
[8] Ivaska, A. (1974), *Talanta,* **21**, 1167-1173
[9] Ivaska, A. and Nagypal, I. (1980), *Magy Kem Foly,* **86**, 2, 84-88
[10] Inczédy, J. (1976), Analytical Application of Complex Equilibria. Ellis Horwood, Chichester

### Evaluation of the Technicon Star system for the estimation of serum $T_4$ and $T_3$-uptake

**S. G. Welshman, M. D. McMaster and T. Linton**
*Clinical Chemistry Department, The Laboratories, Belfast City Hospital, Belfast, Northern Ireland.*

A Technicon Star system supplied by Technicon Instruments Ltd, (Basingstoke, Hampshire, England) was installed to provide a fully automated process for the estimation of serum thyroxine ($T_4$) and $T_3$ -uptake by radioimmunoassay (RIA). The $T_3$-uptake test is a technique used for assessing unsaturated thyroxine-binding globulin capacity in serum by measuring the uptake of radioactive tri-iodothyronine ($T_3$). A daily workload of approximately 150 serum $T_4$ assays and a similar number of $T_3$-uptake tests had previously been estimated by manual radioimmunoassay techniques. An evaluation of the Star system for the estimation of serum $T_4$ and $T_3$-uptake is described.

### Technicon Star system

A continuous flow analyser with computerised control and print-out was used for the automation of radioimmunoassays. The Star RIA system involves competition for antibody sites in the solid-phase reagent between the radio-labelled antigen and the antigen in the sample. The amount of radio-labelled antigen bound to the solid-phase reagent is inversely proportional to the amount of antigen in the sample. As the solid-phase reagent contains ferric oxide particles, the solid-phase bound antigen is retained by activated magnets and the unbound antigen removed to waste. When the magnetic field is removed, the bound radio-labelled antigen is released for measurement in a gamma counter. As the analyser is online to a laboratory computer, the results of both $T_4$ and $T_3$-uptake tests on each serum sample are fed directly to the computer which then calculates the free thyroxine index (FTI) and prints the final report. The reagents for these analyses were originally purchased from Technia, however the company has ceased to operate and all kits are now supplied by Technicon. All results referred to in this paper were obtained using the latter kits.

